# C-Peptide Changes and Glycemic Response During DPP-4 Versus SGLT2 Inhibitor Therapy in Type 2 Diabetes: The Role of Baseline C-Peptide

**DOI:** 10.3390/medicina62101934

**Published:** 2026-10-07

**Authors:** İrfan Alişan, Kubilay Akbal, Çisem Yılmaz, Cahit Dinçer, Bektaş Işık, Ahmet Gazi Mustan, Fatih Necip Arıcı, Hüseyin Ali Öztürk, Hilmi Erdem Sümbül

**Affiliations:** 1 Department of Internal Medicine, Adana City Training and Research Hospital, Adana 01230, Türkiye; 2 Department of Internal Medicine, 5 Ocak State Hospital, Adana 01210, Türkiye

**Keywords:** type 2 diabetes mellitus, C-peptide, DPP-4 inhibitors, SGLT2 inhibitors, glycemic response

## Abstract

*Background and Objective:* To compare longitudinal changes in serum C-peptide during dipeptidyl peptidase-4 (DPP-4) inhibitor versus sodium–glucose cotransporter 2 (SGLT2) inhibitor therapy in type 2 diabetes and to evaluate whether baseline C-peptide predicts glycemic response. *Materials and Methods:* This single-center retrospective new-user cohort study included 196 adults with type 2 diabetes who initiated a DPP-4 inhibitor (*n* = 99) or an SGLT2 inhibitor (*n* = 97) following a 12-month washout period. Baseline and follow-up HbA1c, plasma glucose, and fasting C-peptide measurements were assessed over a mean follow-up of 156 days. Glycemic response was defined as an HbA1c reduction of at least 1.0 percentage point. Multivariable linear and logistic regression analyses were performed, and receiver operating characteristic analysis evaluated the predictive performance of baseline C-peptide. *Results:* HbA1c decreased significantly in both groups, with no between-group difference in HbA1c reduction (1.03 ± 0.87 vs. 0.97 ± 0.83 percentage points; *p* = 0.650). C-peptide increased significantly in the DPP-4 inhibitor group but not in the SGLT2 inhibitor group (0.18 ± 0.44 vs. 0.03 ± 0.38 ng/mL; between-group *p* = 0.010). DPP-4 inhibitor therapy remained independently associated with higher follow-up C-peptide (adjusted β = 0.149; *p* = 0.013). Baseline C-peptide independently predicted glycemic response (adjusted OR = 2.58 per 0.5 ng/mL increase; *p* < 0.001), whereas treatment class and the treatment-by-C-peptide interaction were not significant. Baseline C-peptide showed good discrimination for response (AUC = 0.805), with a Youden-derived exploratory cut-off of 2.33 ng/mL. *Conclusions:* DPP-4 inhibitors were associated with a greater increase in fasting C-peptide than SGLT2 inhibitors despite similar glycemic improvement; however, fasting C-peptide change alone should not be interpreted as direct evidence of improved or preserved β-cell function. Higher baseline C-peptide was associated with glycemic response in both treatment groups, although external validation is required before clinical application.

## 1. Introduction

Type 2 diabetes mellitus is characterized by insulin resistance together with progressive deterioration of pancreatic β-cell function, resulting in insufficient insulin secretion and worsening glycemic control over time [1,2]. Because insulin undergoes substantial and variable hepatic first-pass clearance, circulating insulin concentrations may not accurately reflect endogenous secretion [3]. C-peptide is secreted in equimolar amounts with endogenous insulin and has therefore become a practical marker of residual β-cell secretory capacity in patients with diabetes [4,5]. However, fasting C-peptide is a static measure influenced by the prevailing glycemic state, insulin resistance, renal clearance, and secretory demand; therefore, longitudinal changes in fasting C-peptide should not be interpreted as direct evidence of β-cell recovery, preservation, or structural modification. Assessment of C-peptide may provide clinically relevant information beyond HbA1c by reflecting the underlying capacity to respond to glucose-lowering therapies.

Dipeptidyl peptidase-4 inhibitors and sodium–glucose cotransporter 2 inhibitors are widely used oral glucose-lowering agents with distinct mechanisms of action [6,7]. DPP-4 inhibitors prolong endogenous incretin activity and enhance glucose-dependent insulin secretion while suppressing inappropriate glucagon release [7,8]. In contrast, SGLT2 inhibitors reduce plasma glucose primarily by increasing urinary glucose excretion through an insulin-independent mechanism [9,10]. Although both drug classes improve glycemic control with a relatively low intrinsic risk of hypoglycemia, SGLT2 inhibitors additionally provide established cardiovascular and renal benefits in selected patients, whereas DPP-4 inhibitors are generally weight neutral and are commonly used when treatment simplicity and tolerability are prioritized [9].

Despite their different primary mechanisms, both drug classes may influence β-cell function [11,12]. DPP-4 inhibition may enhance endogenous insulin secretion in individuals with preserved β-cell reserve, and previous studies have suggested that baseline C-peptide may be associated with the glycemic response to DPP-4 inhibitor therapy [13]. SGLT2 inhibition may indirectly improve β-cell function by reducing glucotoxicity and lowering the secretory burden placed on pancreatic β-cells [14]. However, direct comparative evidence regarding longitudinal changes in C-peptide during DPP-4 inhibitor versus SGLT2 inhibitor therapy remains limited, and it is not fully established whether baseline C-peptide identifies patients who are more likely to achieve a favorable glycemic response irrespective of treatment class.

The present study aimed to compare changes in serum C-peptide levels during DPP-4 inhibitor and SGLT2 inhibitor therapy in patients with type 2 diabetes. We additionally evaluated changes in HbA1c and plasma glucose, investigated the association between baseline C-peptide and glycemic response, and assessed whether the relationship between baseline C-peptide and treatment response differed according to the glucose-lowering drug class.

## 2. Materials and Methods

### 2.1. Study Design and Setting

This single-center, retrospective cohort study was conducted in the Department of Internal Medicine at Adana City Training and Research Hospital, Adana, Türkiye. The overall study period extended from January 2024 through May 2026. The study was based on routinely collected electronic medical records and electronic prescribing data (Akgün Hastane Bilgi Yönetim Sistemi, version 26.4.240, Akgün Yazılım, Ankara, Türkiye) of adult patients with established type 2 diabetes. Eligible treatment initiations occurred between January 2024 and December 2025, and follow-up data were collected through May 2026. Eligible treatment initiation was defined as the first prescription of a DPP-4 inhibitor or an SGLT2 inhibitor following a 12-month washout period during which no prescription for either drug class was recorded. The date of this prescription was designated as the index date. Baseline data were obtained from measurements recorded closest to the index date within the preceding 30 days, whereas follow-up assessments were obtained 105–210 days after the index date.

### 2.2. Participants and Eligibility Criteria

Consecutive patients aged ≥18 years with an established diagnosis of type 2 diabetes who initiated a DPP-4 inhibitor or an SGLT2 inhibitor were evaluated. Patients were required to have at least 12 months of available prescription history before the index date. New use was defined as the absence of any recorded prescription for a DPP-4 inhibitor or an SGLT2 inhibitor during this 12-month pre-index washout period. Type 2 diabetes was identified from specialist documentation and clinical records consistent with accepted diagnostic criteria, including HbA1c ≥ 6.5%, fasting plasma glucose ≥126 mg/dL, 2-h plasma glucose ≥200 mg/dL during an oral glucose tolerance test, or random plasma glucose ≥200 mg/dL in the presence of typical hyperglycemic symptoms. Patients were eligible when baseline and follow-up HbA1c and C-peptide measurements were available, plasma glucose was measured on the same day as C-peptide, baseline renal function was documented, and the index treatment was continued throughout the follow-up period. Background glucose-lowering therapy was required to remain unchanged in both medication class and dose throughout follow-up; patients with any initiation, discontinuation, or dose adjustment of concomitant glucose-lowering therapy were excluded.

Patients with type 1 diabetes, latent autoimmune diabetes in adults, gestational diabetes, secondary diabetes, previous pancreatic surgery, chronic pancreatitis, pregnancy, kidney replacement therapy, or an estimated glomerular filtration rate <30 mL/min/1.73 m^2^ were excluded. Patients were also excluded in the presence of missing laboratory data, systemic glucocorticoid use, acute infection, hyperglycemic crisis, major hospitalization, discontinuation or switching of the index drug.

### 2.3. Treatment Exposure and Data Collection

Patients were categorized according to the glucose-lowering drug class prescribed on the index date. The DPP-4 inhibitor group included patients receiving sitagliptin, linagliptin, vildagliptin, saxagliptin, or alogliptin, whereas the SGLT2 inhibitor group included those receiving dapagliflozin or empagliflozin. Treatment class, treatment initiation date, and concomitant glucose-lowering medications were ascertained from the electronic prescribing and medical record systems. Demographic and clinical variables included age, sex, body mass index, diabetes duration, atherosclerotic cardiovascular disease, heart failure, renal function, and the use of metformin, insulin, and sulfonylureas. Body mass index was calculated as weight in kilograms divided by height in meters squared. Laboratory and clinical data were extracted using a standardized data-collection form, and implausible or internally inconsistent values were verified against the original medical records.

### 2.4. Laboratory Measurements

Blood samples for C-peptide measurement were obtained after an overnight fast of at least 10 h. A plasma glucose measurement performed on the same day was required to account for the prevailing glycemic state. Baseline and follow-up C-peptide measurements were obtained under comparable sampling conditions and analyzed using the same method. Serum C-peptide was measured using the Elecsys C-Peptide electrochemiluminescence immunoassay on the cobas e801 platform (Roche Diagnostics GmbH, Mannheim, Germany), with an analytical measuring range of 0.02–40 ng/mL. The manufacturer-reported repeatability and intermediate-precision coefficients of variation ranged from 0.9% to 2.9% and from 2.3% to 3.6%, respectively. HbA1c was measured in ethylenediaminetetraacetic acid-anticoagulated whole blood by cation-exchange high-performance liquid chromatography using the VARIANT II TURBO Hemoglobin Testing System (Bio-Rad Laboratories, Hercules, CA, USA). The assay was certified by the National Glycohemoglobin Standardization Program and traceable to the International Federation of Clinical Chemistry reference method. Plasma glucose was measured using the hexokinase method, whereas serum creatinine was measured using an isotope-dilution mass-spectrometry-traceable enzymatic method on the cobas c702 platform (Roche Diagnostics GmbH, Mannheim, Germany). Estimated glomerular filtration rate was calculated using the 2021 CKD-EPI creatinine equation and expressed as mL/min/1.73 m^2^. All laboratory analyses were performed in the hospital’s central laboratory according to the manufacturers’ instructions and routine internal quality-control procedures.

### 2.5. Study Outcomes

The primary outcome was the change in serum C-peptide concentration from baseline to follow-up, calculated as the follow-up value minus the baseline value. The primary comparison evaluated whether C-peptide change differed between the DPP-4 inhibitor and SGLT2 inhibitor groups. Secondary outcomes included changes in HbA1c and plasma glucose and the proportion of patients achieving a glycemic response. HbA1c and glucose changes were calculated as baseline minus follow-up values, with positive values indicating improvement. Glycemic response was defined as an HbA1c reduction of ≥1.0 percentage point. The association between baseline C-peptide and glycemic response and whether this relationship differed according to treatment class were also evaluated. The unadjusted primary comparison was based on the between-group difference in C-peptide change. For the covariate-adjusted analysis, follow-up C-peptide concentration was modeled as the dependent variable, with baseline C-peptide and prespecified clinical covariates included in the model.

### 2.6. Ethical Approval

The study was approved by the Scientific Research Ethics Committee of Adana City Training and Research Hospital on 21 May 2026 (Meeting No. 26; Decision No. 1354). The study was conducted in accordance with the principles of the Declaration of Helsinki, and all records were de-identified before analysis. The requirement for written informed consent was waived owing to the retrospective nature of the study.

### 2.7. Statistical Analysis

Statistical analyses were performed using IBM SPSS Statistics for Windows, version 27.0 (IBM Corp., Armonk, NY, USA). Normality was assessed using the Kolmogorov–Smirnov and Shapiro–Wilk tests and visual inspection of histograms. Continuous variables are presented as the mean ± standard deviation or median (interquartile range), and categorical variables as number (percentage). Between-group comparisons were performed using Welch’s *t* test, the Mann–Whitney U test, or Fisher’s exact test, as appropriate. Within-group changes were evaluated using paired-samples *t* tests. Baseline covariate balance was assessed using absolute standardized mean differences. Multicollinearity in the multivariable linear and logistic regression models was assessed using variance inflation factors (VIFs). Multivariable linear regression with HC3 robust standard errors was used to evaluate the association between treatment class and follow-up C-peptide concentration after adjustment for baseline C-peptide, baseline glucose, age, sex, BMI, diabetes duration, eGFR, insulin use, and follow-up duration. A sensitivity analysis additionally included same-day follow-up glucose in the model. Multivariable logistic regression was used to identify predictors of glycemic response. A treatment group × baseline C-peptide interaction term was tested. A post hoc sensitivity analysis compared patients receiving neither insulin nor sulfonylureas with those receiving insulin and/or sulfonylureas. The discriminative performance of baseline C-peptide for glycemic response was compared between these strata, and longitudinal C-peptide change was also evaluated according to background therapy; interaction terms were tested to assess potential effect modification. ROC analysis was performed to assess the discriminative ability of baseline C-peptide, and a cut-off maximizing the Youden index was determined. AUC confidence intervals and subgroup AUC differences were estimated using 3000 bootstrap resamples. Analyses were performed using complete cases, and no missing-value imputation was applied. All tests were two sided, and *p* < 0.05 was considered statistically significant.

## 3. Results

A total of 196 eligible new users were included, of whom 99 (50.5%) initiated a DPP-4 inhibitor and 97 (49.5%) initiated an SGLT2 inhibitor. The mean age was 59.6 ± 7.9 years, 51.5% of the cohort was female, and the mean BMI was 31.0 ± 4.3 kg/m^2^. The median duration of diabetes was 8.6 years [interquartile range, 5.7–13.5], and the mean follow-up duration was 156.0 ± 22.5 days, with a median of 157 days [interquartile range, 139–173]. Mean HbA1c decreased from 8.77 ± 0.95% at baseline to 7.77 ± 1.05% at follow-up, corresponding to a mean reduction of 1.00 ± 0.85 percentage points. Overall, 102 participants (52.0%) met the predefined criterion for glycemic response. Median C-peptide increased from 2.38 [1.90–3.12] ng/mL at baseline to 2.49 [2.00–3.21] ng/mL at follow-up (Table 1).

The DPP-4 and SGLT2 inhibitor groups were broadly comparable in terms of demographic, clinical, treatment-related, and baseline laboratory characteristics. There were no statistically significant differences between the groups in age, sex, BMI, diabetes duration, eGFR, cardiovascular comorbidities, background glucose-lowering therapies, baseline HbA1c, baseline C-peptide, baseline glucose, or follow-up duration. Absolute standardized mean differences were generally small, although minor residual imbalances were observed for BMI, diabetes duration, eGFR, baseline C-peptide, and follow-up duration (Table 2).

HbA1c decreased significantly within both treatment groups. The mean HbA1c reduction was 1.03 ± 0.87 percentage points in the DPP-4 inhibitor group and 0.97 ± 0.83 percentage points in the SGLT2 inhibitor group. The difference in HbA1c reduction between the groups was not statistically significant (mean difference, 0.06 percentage points; 95% CI, −0.18 to 0.29; *p* = 0.650). C-peptide increased significantly in the DPP-4 inhibitor group, from a median of 2.34 [1.85–3.04] ng/mL at baseline to 2.57 [2.01–3.25] ng/mL at follow-up, with a mean change of 0.18 ± 0.44 ng/mL (*p* < 0.001). In the SGLT2 inhibitor group, the corresponding change was 0.03 ± 0.38 ng/mL and was not statistically significant (*p* = 0.491). The increase in C-peptide was significantly greater in the DPP-4 inhibitor group than in the SGLT2 inhibitor group (mean difference, 0.15 ng/mL; 95% CI, 0.04–0.27; *p* = 0.010). Glucose levels decreased significantly in both groups; however, the magnitude of glucose reduction did not differ significantly between the treatment groups (mean difference, 1.5 mg/dL; 95% CI, −6.2 to 9.2; *p* = 0.702) (Table 3).

In the unadjusted comparisons, glycemic responders had a shorter median duration of diabetes than nonresponders (7.6 [4.8–10.8] vs. 11.5 [6.3–16.1] years; *p* < 0.001). Responders also had higher mean eGFR (93.8 ± 11.6 vs. 89.4 ± 11.2 mL/min/1.73 m^2^; *p* = 0.008), higher baseline HbA1c (9.00 ± 0.96% vs. 8.52 ± 0.88%; *p* < 0.001), and higher baseline glucose levels (207.1 ± 29.3 vs. 196.2 ± 29.2 mg/dL; *p* = 0.009). Baseline C-peptide was substantially higher among responders than nonresponders (2.92 [2.36–3.51] vs. 2.00 [1.67–2.33] ng/mL; *p* < 0.001). Treatment-group distribution, age, sex, BMI, background glucose-lowering therapy, cardiovascular comorbidities, and follow-up duration did not differ significantly according to glycemic-response status (Table 4).

In the multivariable linear regression model, DPP-4 inhibitor therapy was independently associated with a 0.149-ng/mL higher follow-up C-peptide concentration compared with SGLT2 inhibitor therapy after adjustment for baseline C-peptide, baseline glucose, age, sex, BMI, diabetes duration, eGFR, insulin use, and follow-up duration (adjusted β = 0.149; 95% CI, 0.032–0.267; *p* = 0.013). Baseline C-peptide was also independently associated with follow-up C-peptide concentration (adjusted β = 0.909 per 1-ng/mL increase; 95% CI, 0.835–0.984; *p* < 0.001). Baseline eGFR was not independently associated with follow-up C-peptide (β = −0.010 per 10 mL/min/1.73 m^2^; *p* = 0.728). In a post hoc analysis stratified by baseline eGFR (<90 vs. ≥90 mL/min/1.73 m^2^), the DPP-4–SGLT2 difference in C-peptide change was 0.21 and 0.10 ng/mL, respectively, with no evidence of treatment-by-eGFR stratum interaction (*p* = 0.362). The association remained significant in a sensitivity analysis additionally adjusted for same-day follow-up glucose (adjusted β = 0.144; 95% CI, 0.025–0.263; *p* = 0.018). Same-day follow-up glucose was not independently associated with follow-up C-peptide in this model (adjusted β = −0.016 per 10-mg/dL increase; 95% CI, −0.050–0.017; *p* = 0.343). The model explained a substantial proportion of the variation in follow-up C-peptide concentration (adjusted R^2^ = 0.800) (Table 5).

Higher baseline C-peptide was independently associated with an increased likelihood of glycemic response. Each 0.5-ng/mL increase in baseline C-peptide was associated with a 2.58-fold increase in the odds of response (adjusted OR = 2.58; 95% CI, 1.81–3.68; *p* < 0.001). Higher baseline HbA1c was also independently associated with glycemic response (adjusted OR = 2.98 per 1-percentage-point increase; 95% CI, 1.90–4.67; *p* < 0.001). Treatment class itself was not independently associated with glycemic response (adjusted OR = 1.01; 95% CI, 0.50–2.06; *p* = 0.975). The treatment group × baseline C-peptide interaction was not statistically significant (adjusted OR = 1.27; 95% CI, 0.70–2.32; *p* = 0.432), indicating no evidence that the association between baseline C-peptide and glycemic response differed between the two treatment groups. The complete multivariable model had an AUC of 0.872, a McFadden pseudo-R^2^ of 0.348, and a Brier score of 0.144 (Table 6).

Baseline C-peptide alone showed good discrimination for glycemic response in the overall cohort, with an AUC of 0.805 (95% CI, 0.741–0.864; *p* < 0.001). The Youden-derived exploratory cut-off was 2.33 ng/mL, yielding a sensitivity of 79.4% and a specificity of 74.5%. The corresponding positive and negative predictive values were 77.1% and 76.9%, respectively. The AUC was 0.822 (95% CI, 0.729–0.900) in the DPP-4 inhibitor subgroup and 0.786 (95% CI, 0.690–0.874) in the SGLT2 inhibitor subgroup. The difference between the subgroup AUCs was not statistically significant (AUC difference = 0.036; 95% CI, −0.089 to 0.162; *p* = 0.576) (Table 7).

In a post hoc analysis, 105 patients were receiving neither insulin nor sulfonylureas and 91 were receiving insulin and/or sulfonylureas. Baseline C-peptide showed similar discrimination for glycemic response in the two strata (AUC = 0.825 [95% CI, 0.742–0.898] vs. 0.779 [95% CI, 0.680–0.873]; AUC difference = 0.046, 95% CI −0.077 to 0.168; *p* = 0.492), with no significant baseline C-peptide-by-background-therapy interaction (*p* = 0.230). Mean C-peptide change was also similar between patients receiving neither medication and those receiving insulin and/or sulfonylureas (0.13 ± 0.42 vs. 0.07 ± 0.41 ng/mL; *p* = 0.262), with no evidence of treatment-by-background-therapy interaction (*p* = 0.144).

## 4. Discussion

In this retrospective cohort study, DPP-4 inhibitor and SGLT2 inhibitor therapies were associated with comparable reductions in HbA1c and plasma glucose over approximately five months, while patterns of fasting C-peptide change differed between the treatment groups. C-peptide increased significantly during DPP-4 inhibitor therapy, and the increase was greater than that observed during SGLT2 inhibitor therapy. Although statistically significant, the absolute between-group difference in C-peptide change was modest (0.15 ng/mL) and should be interpreted cautiously, particularly in view of the comparable HbA1c reductions observed in the two treatment groups. This difference remained significant after adjustment for baseline C-peptide, prevailing glucose concentrations, demographic characteristics, diabetes duration, renal function, insulin use, and follow-up duration. In contrast, treatment class was not independently associated with the predefined glycemic response, indicating that the greater C-peptide increase with DPP-4 inhibition did not translate into a superior HbA1c response during the observation period. Baseline C-peptide was instead a strong and treatment-independent predictor of glycemic response, with good discriminatory performance and no significant interaction with drug class. Collectively, these findings indicate comparable short-term glycemic improvement across treatment groups despite different patterns of fasting C-peptide change, while higher baseline C-peptide was associated with a greater likelihood of glycemic response. The present study therefore contributes clinically relevant comparative evidence by integrating longitudinal C-peptide measurements with glycemic outcomes rather than evaluating HbA1c alone.

The greater C-peptide increase observed with DPP-4 inhibitor therapy is biologically consistent with the incretin-dependent mechanism of this drug class. Liu et al. (2024) evaluated patients with newly diagnosed type 2 diabetes and marked hyperglycemia who received short-term intensive insulin therapy followed by linagliptin, metformin, their combination, or lifestyle intervention [15]. Linagliptin-containing treatment, particularly linagliptin combined with metformin, was associated with sustained glycemic control and greater improvement in β-cell function indices [15]. Similarly, in the GRADE cohort, Utzschneider et al. (2025) reported that sitagliptin improved model-derived β-cell glucose sensitivity, although its effects on other secretory parameters were more modest and β-cell function subsequently declined across all treatment groups [16]. These observations are biologically consistent with the greater increase in fasting C-peptide observed in the DPP-4 inhibitor group. Importantly, fasting C-peptide is a static marker of endogenous insulin secretion and should not be considered equivalent to β-cell secretory capacity, which is more comprehensively assessed using model-based or dynamic measures such as HOMA2-%B, mixed-meal tolerance testing, or the disposition index. Fasting C-peptide is influenced by ambient glucose, insulin resistance, renal clearance, and secretory demand; therefore, the observed increase should not be interpreted as direct evidence of β-cell preservation or increased β-cell mass. Our sensitivity analysis adjusting for same-day follow-up glucose reduces the likelihood that the observed difference was explained solely by differences in prevailing glycemia, but longer prospective studies using dynamic stimulation tests are required to determine whether the effect represents improved β-cell responsiveness, increased basal secretion, or a temporary pharmacodynamic response.

The absence of a significant C-peptide increase in the SGLT2 inhibitor group does not necessarily indicate an absence of beneficial effects on β-cell physiology. Triplitt et al. (2025) found that dapagliflozin monotherapy produced sustained improvements in model-based β-cell function and insulin sensitivity in patients with type 2 diabetes, despite its primary insulin-independent mechanism of action [17]. More recently, Thirumathyam et al. (2026) reported in a randomized crossover study that empagliflozin improved β-cell glucose sensitivity and disposition-index measures compared with glycemia-matched insulin treatment, suggesting that some β-cell effects of SGLT2 inhibition may extend beyond the simple relief of glucotoxicity [18]. Our finding of essentially unchanged fasting C-peptide during SGLT2 inhibitor therapy may therefore reflect a reduction in the insulin secretory burden rather than a failure to improve β-cell function. By promoting urinary glucose excretion, SGLT2 inhibitors lower plasma glucose without requiring increased insulin output; consequently, improved glucose sensitivity or insulin secretion relative to insulin resistance can coexist with stable or even reduced absolute C-peptide concentrations. This distinction may explain why HbA1c and glucose decreased substantially in our SGLT2 inhibitor group despite the absence of a measurable rise in fasting C-peptide. Accordingly, fasting C-peptide change and dynamic β-cell function should be regarded as related but non-equivalent outcomes.

The strong association between baseline C-peptide and glycemic response was another central finding of our study. Each 0.5-ng/mL increase in baseline C-peptide was associated with a 2.58-fold increase in the adjusted odds of achieving an HbA1c reduction of at least 1 percentage point. Baseline C-peptide alone showed good discrimination, with an AUC of 0.805 and a Youden-derived cut-off of 2.33 ng/mL. However, responders also had higher baseline HbA1c, and baseline HbA1c remained independently associated with glycemic response in the multivariable model, indicating that the observed response cannot be attributed to baseline C-peptide alone. Yoshiji et al. (2022) similarly demonstrated that fasting and meal-stimulated C-peptide measures were associated with the HbA1c response to dulaglutide [19]. Fasting C-peptide predicted an HbA1c reduction of at least 1 percentage point with an AUC of 0.83, which is remarkably close to the discrimination observed in our cohort [19]. Enkaku et al. (2024) further showed that short-term recovery of meal-stimulated insulin secretion, assessed using C-peptide-based indices, was associated with subsequent HbA1c control and more favorable continuous-glucose-monitoring metrics [20]. Our results extend these observations by demonstrating that the predictive value of baseline C-peptide was not restricted to an incretin-based treatment. The absence of a significant treatment-by-C-peptide interaction and the similar subgroup AUCs indicate that higher baseline C-peptide was similarly associated with glycemic improvement during both DPP-4 and SGLT2 inhibitor therapy. Baseline C-peptide may therefore represent a general marker of the capacity to benefit from non-insulin glucose-lowering treatment rather than a class-specific companion biomarker.

The predictive role of C-peptide also emphasizes the biological heterogeneity concealed within the clinical diagnosis of type 2 diabetes. Fan et al. (2023) showed in a large cohort of patients diagnosed with type 2 diabetes that C-peptide and GAD-antibody status identified clinically distinct phenotypes with different patterns of insulin initiation, glycemic response, and severe hypoglycemia, supporting more detailed characterization of β-cell dysfunction in routine diabetes care [21]. In a contemporary review, Lin et al. (2025) likewise proposed that C-peptide could support diabetes classification, assessment of endogenous insulin secretion, treatment selection, and prediction of therapeutic outcomes, while emphasizing that its interpretation must account for concurrent glucose concentrations, renal function, insulin resistance, and clinical context [22]. Our findings support this precision-treatment framework because baseline C-peptide provided information beyond baseline HbA1c and treatment class. Established C-peptide thresholds serve a different clinical purpose from the cut-off identified in the present study. Values around 0.6–1.8 ng/mL are primarily used in the assessment of diabetes classification and insulin deficiency, with substantial diagnostic overlap within this range, whereas values above approximately 1.8 ng/mL generally support preserved endogenous insulin secretion and, in the appropriate clinical context, type 2 diabetes [22]. In contrast, our fasting C-peptide cut-off of 2.33 ng/mL was derived solely to discriminate glycemic response within a cohort of patients with established type 2 diabetes and should not be interpreted as a diagnostic threshold or a boundary for insulin requirement. Its numerical difference from established classification thresholds reflects the different population, sampling context, and clinical endpoint. Although all C-peptide measurements in the present study were performed using the same assay, between-assay differences have been reported across C-peptide measurement platforms; therefore, the numerical cut-off of 2.33 ng/mL should not be directly extrapolated to laboratories using different assay methods without assay-specific validation [22]. Moreover, because this cut-off was derived and evaluated in the same cohort, it should be regarded as hypothesis generating and requires external validation before being used for treatment selection.

The comparable HbA1c reductions in the two groups indicate that the greater C-peptide increase during DPP-4 inhibitor therapy should not be interpreted as evidence of overall therapeutic superiority. In a large new-user study, D’Andrea et al. (2023) found that SGLT2 inhibitor initiation was associated with lower risks of major cardiovascular events, heart-failure hospitalization, and acute kidney injury than DPP-4 inhibitor initiation, although genital infections and diabetic ketoacidosis were more frequent; these comparative associations were generally consistent across baseline HbA1c categories [23]. Consistently, the clinical guideline developed by Qaseem et al. (2024) prioritized SGLT2 inhibitors in patients requiring additional cardiorenal risk reduction because their benefits extend beyond glucose lowering, whereas DPP-4 inhibitors were not associated with comparable reductions in morbidity or mortality [24]. Thus, C-peptide response represents only one dimension of treatment evaluation, and drug selection should continue to incorporate cardiovascular disease, heart failure, chronic kidney disease, hypoglycemia risk, tolerability, cost, and patient preference. Our results must also be interpreted in view of the single-center retrospective design, absence of random treatment allocation, potential differences among individual agents within each class, and the possibility of residual confounding despite multivariable adjustment. Furthermore, fasting C-peptide was measured at only two time points, and dynamic β-cell function, urinary glucose excretion, insulin sensitivity, adherence, dietary changes, and weight change were not directly assessed. Prospective studies with standardized stimulation testing and longer follow-up are needed to confirm whether the observed C-peptide divergence persists and whether baseline C-peptide can meaningfully improve individualized treatment selection.

This study has several limitations. Its retrospective, single-center design precludes causal inference and may limit the generalizability of the findings to populations with different demographic, clinical, or treatment characteristics. Because treatment allocation was not randomized, confounding by indication and residual confounding may have persisted despite the broad similarity of the groups at baseline and multivariable adjustment for relevant clinical variables. Fasting C-peptide was measured at only two time points over a relatively short follow-up period, and dynamic assessments of β-cell function, such as glucagon-stimulated or mixed-meal tolerance testing, were not available. Therefore, changes in fasting C-peptide cannot be interpreted as direct evidence of β-cell preservation or changes in β-cell mass. Although same-day plasma glucose was considered and the sensitivity analysis additionally adjusted for follow-up glucose, C-peptide concentrations may also have been influenced by insulin resistance, dietary intake, renal clearance, weight change, and other unmeasured physiological factors. Concomitant insulin and sulfonylurea therapy may also influence fasting C-peptide concentrations independently of the index drug class; although background therapy was required to remain stable and post hoc analyses showed no significant effect modification, residual influence from these treatments cannot be excluded. Treatment adherence, urinary glucose excretion, changes in insulin sensitivity, and lifestyle modifications were not systematically quantified. Moreover, the requirement for complete baseline and follow-up measurements may have introduced selection bias, while the sample size may have limited the statistical power to detect modest treatment-by-C-peptide interactions or differences between individual drugs. Patient-level identity and dose information for individual DPP-4 and SGLT2 inhibitors were not retained in the final analytic dataset; therefore, agent-specific patient counts, dose distributions, and potential heterogeneity in C-peptide response among individual drugs could not be evaluated reliably. Follow-up eGFR was not systematically available, precluding direct assessment of the association between longitudinal changes in renal function and C-peptide; however, baseline eGFR was included in the adjusted model, and the stratified analysis showed no evidence of effect modification by baseline renal function. Finally, the C-peptide cut-off identified by ROC analysis was derived and evaluated in the same cohort and should be externally validated before being applied in clinical decision-making.

## 5. Conclusions

In conclusion, DPP-4 inhibitor and SGLT2 inhibitor therapies were associated with comparable reductions in HbA1c and plasma glucose, whereas DPP-4 inhibitor therapy was associated with a significantly greater increase in fasting C-peptide. This association remained significant after adjustment for baseline C-peptide, baseline glucose, demographic and clinical characteristics, renal function, insulin use, follow-up duration, and same-day follow-up glucose. In contrast, treatment class was not independently associated with the likelihood of achieving an HbA1c reduction of at least 1 percentage point. Baseline C-peptide emerged as a strong and treatment-independent predictor of glycemic response, with good discriminatory performance in both treatment groups. These findings indicate similar short-term glycemic improvement across treatment groups despite different patterns of fasting C-peptide change and suggest that baseline C-peptide may provide clinically relevant information when estimating treatment response. Prospective, multicenter studies incorporating standardized dynamic β-cell function tests, longer follow-up, and external validation of the exploratory C-peptide cut-off are required to confirm these findings and clarify the potential role of C-peptide in individualized glucose-lowering therapy.

## Figures and Tables

**Table 1:** Overall Characteristics and Study Outcomes of the Cohort.

Characteristic	Overall Cohort (N = 196)
**Treatment distribution**	
DPP-4 inhibitor, *n* (%)	99 (50.5)
SGLT2 inhibitor, *n* (%)	97 (49.5)
**Demographic and clinical characteristics**	
Age, years	59.6 ± 7.9
Female sex, *n* (%)	101 (51.5)
BMI, kg/m^2^	31.0 ± 4.3
Diabetes duration, years	8.6 [5.7–13.5]
eGFR, mL/min/1.73 m^2^	91.7 ± 11.6
ASCVD, n (%)	17 (8.7)
Heart failure, *n* (%)	7 (3.6)
**Background glucose-lowering therapy**	
Metformin use, *n* (%)	153 (78.1)
Insulin use, *n* (%)	52 (26.5)
Sulfonylurea use, *n* (%)	50 (25.5)
**Glycemic and C-peptide measures**	
Baseline HbA1c, %	8.77 ± 0.95
Follow-up HbA1c, %	7.77 ± 1.05
HbA1c reduction, percentage points	1.00 ± 0.85
Glycemic responders, *n* (%)	102 (52.0)
Baseline C-peptide, ng/mL	2.38 [1.90–3.12]
Follow-up C-peptide, ng/mL	2.49 [2.00–3.21]
C-peptide change, ng/mL	0.10 ± 0.41
Baseline glucose, mg/dL	201.9 ± 29.7
Follow-up glucose, mg/dL	172.9 ± 35.0
Follow-up duration, days	156.0 ± 22.5

Data are presented as the mean ± standard deviation, median [interquartile range], or number (percentage), as appropriate. Glycemic response was defined as an HbA1c reduction of ≥1.0 percentage point from baseline. ASCVD, atherosclerotic cardiovascular disease; BMI, body mass index; DPP-4, dipeptidyl peptidase-4; eGFR, estimated glomerular filtration rate; HbA1c, glycated hemoglobin; SGLT2, sodium–glucose cotransporter 2.

**Table 2:** Baseline Characteristics According to Treatment Group.

Characteristic	DPP-4 Inhibitor (*n* = 99)	SGLT2 Inhibitor (*n* = 97)	*p* Value	Absolute SMD
**Age, years**	59.3 ± 7.7	59.9 ± 8.2	0.608	0.073
**Female sex, *n* (%)**	52 (52.5)	49 (50.5)	0.886	0.040
**BMI, kg/m^2^**	30.8 ± 4.1	31.3 ± 4.5	0.373	0.128
**Diabetes duration, years**	8.6 [5.8–13.4]	8.7 [5.3–13.8]	0.764	0.107
**eGFR, mL/min/1.73 m^2^**	92.5 ± 11.8	90.8 ± 11.4	0.320	0.143
**ASCVD, *n* (%)**	10 (10.1)	7 (7.2)	0.613	0.103
**Heart failure, *n* (%)**	4 (4.0)	3 (3.1)	1.000	0.051
**Metformin use, *n* (%)**	79 (79.8)	74 (76.3)	0.607	0.085
**Insulin use, *n* (%)**	28 (28.3)	24 (24.7)	0.629	0.080
**Sulfonylurea use, *n* (%)**	26 (26.3)	24 (24.7)	0.870	0.035
**Baseline HbA1c, %**	8.78 ± 0.97	8.76 ± 0.93	0.895	0.019
**Baseline C-peptide, ng/mL**	2.34 [1.85–3.04]	2.40 [1.91–3.20]	0.549	0.131
**Baseline glucose, mg/dL**	202.0 ± 30.2	201.7 ± 29.4	0.938	0.011
**Follow-up duration, days**	157.5 ± 20.3	154.4 ± 24.5	0.347	0.135

Data are presented as the mean ± standard deviation, median [interquartile range], or number (percentage). Continuous variables were compared using Welch’s t test or the Mann–Whitney U test, and categorical variables using Fisher’s exact test. Absolute SMDs describe baseline covariate balance; no matching or propensity-score method was applied. All *p* values are two sided. ASCVD, atherosclerotic cardiovascular disease; BMI, body mass index; DPP-4, dipeptidyl peptidase-4; eGFR, estimated glomerular filtration rate; HbA1c, glycated hemoglobin; SGLT2, sodium–glucose cotransporter 2; SMD, standardized mean difference.

**Table 3:** Within-Group and Between-Group Changes in Glycemic and C-Peptide Measures.

Outcome	Group	Baseline	Follow-Up	Change	Within-Group *p*	Difference in Change, DPP-4 Minus SGLT2 (95% CI)	Between-Group *p*
**HbA1c, %**	DPP-4 inhibitor	8.78 ± 0.97	7.75 ± 1.10	1.03 ± 0.87	<0.001	+0.06 (−0.18 to 0.29)	0.650
	SGLT2 inhibitor	8.76 ± 0.93	7.79 ± 1.00	0.97 ± 0.83	<0.001		
**C-peptide, ng/mL**	DPP-4 inhibitor	2.34 [1.85–3.04]	2.57 [2.01–3.25]	0.18 ± 0.44	<0.001	+0.15 (0.04 to 0.27)	0.010
	SGLT2 inhibitor	2.40 [1.91–3.20]	2.46 [1.93–3.04]	0.03 ± 0.38	0.491		
**Glucose, mg/dL**	DPP-4 inhibitor	202.0 ± 30.2	172.3 ± 32.9	29.8 ± 28.5	<0.001	+1.5 (−6.2 to 9.2)	0.702
	SGLT2 inhibitor	201.7 ± 29.4	173.4 ± 37.2	28.3 ± 26.0	<0.001		

Data are presented as the mean ± standard deviation or median [interquartile range], as appropriate. Baseline and follow-up C-peptide distributions deviated from normality, whereas C-peptide change scores were approximately normally distributed; accordingly, baseline and follow-up values are reported as median [interquartile range] and changes as the mean ± standard deviation. Within-group *p* values were obtained using paired *t* tests and between-group *p* values using Welch’s *t* tests of change scores. Positive changes indicate reductions in HbA1c and glucose and increases in C-peptide. All *p* values are two sided. CI, confidence interval; DPP-4, dipeptidyl peptidase-4; HbA1c, glycated hemoglobin; SGLT2, sodium–glucose cotransporter 2.

**Table 4:** Clinical Characteristics According to Glycemic Response Status.

Characteristic	Responders (*n* = 102)	Nonresponders (*n* = 94)	*p* Value	Absolute SMD
**DPP-4 inhibitor, *n* (%)**	51 (50.0)	48 (51.1)	0.887	0.021
**Age, years**	59.3 ± 8.0	59.9 ± 7.9	0.596	0.076
**Female sex, *n* (%)**	56 (54.9)	45 (47.9)	0.391	0.141
**BMI, kg/m^2^**	31.3 ± 4.0	30.8 ± 4.6	0.390	0.123
**Diabetes duration, years**	7.6 [4.8–10.8]	11.5 [6.3–16.1]	<0.001	0.618
**eGFR, mL/min/1.73 m^2^**	93.8 ± 11.6	89.4 ± 11.2	0.008	0.384
**Baseline HbA1c, %**	9.00 ± 0.96	8.52 ± 0.88	<0.001	0.517
**Baseline C-peptide, ng/mL**	2.92 [2.36–3.51]	2.00 [1.67–2.33]	<0.001	1.184
**Baseline glucose, mg/dL**	207.1 ± 29.3	196.2 ± 29.2	0.009	0.375
**Metformin use, *n* (%)**	78 (76.5)	75 (79.8)	0.608	0.080
**Insulin use, *n* (%)**	29 (28.4)	23 (24.5)	0.627	0.090
**Sulfonylurea use, *n* (%)**	22 (21.6)	28 (29.8)	0.195	0.188
**ASCVD, *n* (%)**	11 (10.8)	6 (6.4)	0.318	0.157
**Heart failure, *n* (%)**	4 (3.9)	3 (3.2)	1.000	0.039
**Follow-up duration, days**	154.8 ± 22.9	157.3 ± 22.1	0.437	0.111

Data are presented as the mean ± standard deviation, median [interquartile range], or number (percentage). Glycemic response was defined as an HbA1c reduction of ≥1.0 percentage point. Continuous variables were compared using Welch’s t test or the Mann–Whitney U test, and categorical variables using Fisher’s exact test. Comparisons are unadjusted and exploratory. Absolute SMDs describe between-group differences. All *p* values are two sided. ASCVD, atherosclerotic cardiovascular disease; BMI, body mass index; DPP-4, dipeptidyl peptidase-4; eGFR, estimated glomerular filtration rate; HbA1c, glycated hemoglobin; SMD, standardized mean difference.

**Table 5:** Multivariable Linear Regression Analysis of Follow-Up C-Peptide.

Predictor	Adjusted β	95% CI	*p* Value
**DPP-4 vs. SGLT2**	0.149	0.032 to 0.267	0.013
**Baseline C-peptide, per 1 ng/mL**	0.909	0.835 to 0.984	<0.001
**Baseline glucose, per 10 mg/dL**	0.004	−0.014 to 0.023	0.652
**Age, per 10 years**	−0.055	−0.128 to 0.018	0.139
**Female sex**	−0.012	−0.132 to 0.109	0.848
**BMI, per 5 kg/m^2^**	0.030	−0.056 to 0.117	0.489
**Diabetes duration, per 5 years**	0.008	−0.043 to 0.059	0.769
**eGFR, per 10 mL/min/1.73 m^2^**	−0.010	−0.067 to 0.047	0.728
**Insulin use**	−0.038	−0.177 to 0.100	0.589
**Follow-up, per 30 days**	−0.063	−0.145 to 0.019	0.131

The dependent variable was follow-up C-peptide concentration. Unstandardized β coefficients were estimated using HC3 robust standard errors. The model was adjusted for treatment group, baseline C-peptide, baseline glucose, age, sex, BMI, diabetes duration, eGFR, insulin use, and follow-up duration. Reference categories were SGLT2 inhibitor therapy, male sex, and no insulin use. Model N = 196; adjusted R^2^ = 0.800. All *p* values are two sided. The treatment association remained significant in a sensitivity analysis additionally adjusted for same-day follow-up glucose (β = 0.144; 95% CI, 0.025–0.263; *p* = 0.018). VIFs for the model covariates ranged from 1.02 to 1.40, indicating no evidence of problematic multicollinearity. BMI, body mass index; CI, confidence interval; DPP-4, dipeptidyl peptidase-4; eGFR, estimated glomerular filtration rate; SGLT2, sodium–glucose cotransporter 2.

**Table 6:** Multivariable Logistic Regression Analysis of Glycemic Response.

Predictor	Adjusted OR	95% CI	*p* Value
**Baseline C-peptide, per 0.5 ng/mL**	2.58	1.81 to 3.68	<0.001
**DPP-4 inhibitor vs. SGLT2 inhibitor**	1.01	0.50 to 2.06	0.975
**Baseline HbA1c, per 1-percentage-point increase**	2.98	1.90 to 4.67	<0.001
**Age, per 10 years**	1.22	0.77 to 1.94	0.402
**Female sex**	1.20	0.57 to 2.52	0.626
**BMI, per 5 kg/m^2^**	0.96	0.64 to 1.44	0.859
**Diabetes duration, per 5 years**	0.77	0.55 to 1.09	0.145
**eGFR, per 10 mL/min/1.73 m^2^**	1.37	0.97 to 1.95	0.078
**Insulin use**	1.47	0.57 to 3.75	0.423
**Follow-up duration, per 30 days**	0.83	0.52 to 1.32	0.429

Glycemic response was defined as an HbA1c reduction of ≥1.0 percentage point. Adjusted ORs were estimated using multivariable logistic regression including baseline C-peptide, treatment group, baseline HbA1c, age, sex, BMI, diabetes duration, eGFR, insulin use, and follow-up duration. Reference categories were SGLT2 inhibitor therapy, male sex, and no insulin use. Model N = 196, with 102 responders; AUC = 0.872, McFadden pseudo-R^2^ = 0.348, and Brier score = 0.144. The AUC refers to the complete multivariable model. The treatment × baseline C-peptide interaction was not significant (OR = 1.27, 95% CI 0.70–2.32; *p* = 0.432). VIFs for the model covariates ranged from 1.02 to 1.40, indicating no evidence of problematic multicollinearity. BMI, body mass index; CI, confidence interval; DPP-4, dipeptidyl peptidase-4; eGFR, estimated glomerular filtration rate; HbA1c, glycated hemoglobin; OR, odds ratio; SGLT2, sodium–glucose cotransporter 2.

**Table 7:** ROC Analysis of Baseline C-Peptide for Glycemic Response.

Population	AUC (95% CI)	*p* Value	Cut-Off, ng/mL	Sensitivity, %	Specificity, %	PPV, %	NPV, %	LR+/LR−
**Overall cohort**	0.805 (0.741–0.864)	<0.001	2.33	79.4	74.5	77.1	76.9	3.11/0.28
**DPP-4 inhibitor subgroup**	0.822 (0.729–0.900)	<0.001	2.45	74.5	81.2	80.9	75.0	3.97/0.31
**SGLT2 inhibitor subgroup**	0.786 (0.690–0.874)	<0.001	2.34	80.4	71.7	75.9	76.7	2.84/0.27

ROC analysis evaluated baseline C-peptide alone. Cut-offs were determined using the Youden index, and AUC confidence intervals were estimated using 3000 nonparametric bootstrap resamples. *p* values test the null hypothesis that AUC = 0.50; PPV and NPV reflect the observed response prevalence. Subgroup AUCs did not differ significantly (difference = 0.036, 95% CI −0.089 to 0.162; *p* = 0.576). AUC, area under the curve; CI, confidence interval; DPP-4, dipeptidyl peptidase-4; LR, likelihood ratio; NPV, negative predictive value; PPV, positive predictive value; ROC, receiver operating characteristic; SGLT2, sodium–glucose cotransporter 2.

## Data Availability

The datasets generated and/or analyzed during the current study are available from the corresponding author upon reasonable request, subject to institutional and ethical regulations concerning patient confidentiality and data protection.
